# A Global Comparative Genomic Analysis of Major Bacterial Pathogens in Bovine Mastitis and Lameness

**DOI:** 10.3390/ani15030394

**Published:** 2025-01-30

**Authors:** Szilvia Kusza, Zoltán Bagi

**Affiliations:** Centre for Agricultural Genomics and Biotechnology, University of Debrecen, Egyetem tér 1, H-4032 Debrecen, Hungary; bagiz@agr.unideb.hu

**Keywords:** animal health, bovine, genomics, mastitis, lameness

## Abstract

This study presents a comprehensive genomic reanalysis of major bacterial pathogens causing bovine mastitis and lameness, focusing on *Staphylococcus aureus*, *Escherichia coli*, *Fusobacterium necrophorum*, and *Treponema phagedenis*. Through our analysis of 4326 bacterial genomes from global databases, we identified distinct patterns in genomic diversity, virulence factors and antimicrobial resistance genes across these species. *E. coli* showed the highest genomic diversity with 3779 isolates, of which 98% exhibited high-quality genome sequences. Similarly, *S. aureus* demonstrated significant genomic plasticity across 524 isolates, with 99.8% classified as high-quality genomes. Geographical analysis revealed distinct regional variations in strain distribution, with North America contributing 45.3% of all isolates, followed by Asia (21.2%) and Europe (18.1%). Furthermore, we identified novel virulence mechanisms and resistance patterns specific to each pathogen, with particular emphasis on the evolution of antimicrobial resistance genes. Our findings provide crucial insights into pathogen adaptation and host–microbe interactions, suggesting the need for region-specific intervention strategies. These results have significant implications for developing targeted therapeutic approaches and improving bovine health management practices.

## Simple Summary

Dairy cows commonly suffer from two major health problems, mastitis (udder inflammation) and lameness (difficulty walking), which cause significant pain and reduce milk production. These conditions are mainly caused by bacteria; however we do not fully understand how these bacteria adapt and spread. In our work, we focused on the genetic material of four important bacterial species that cause the mentioned diseases, analyzing over 4300 bacterial samples worldwide. We found that these bacteria showed different genomic diversity patterns depending on where they originated, suggesting they adapt to local conditions. Some bacteria also showed signs of becoming resistant to common treatments, which is concerning for animal health. We found that two bacterial species, *Escherichia coli* and *Staphylococcus aureus*, were much more common and varied than the others. These findings help farmers and veterinarians better understand how these diseases spread and develop. This knowledge can lead to better treatments and prevention strategies, ultimately improving cow welfare and dairy farm productivity while reducing economic losses in the dairy industry.

## 1. Introduction

Bovine health challenges, particularly mastitis and lameness, represent critical concerns in global dairy production, causing substantial economic losses and welfare issues worldwide. Global mastitis prevalence shows distinct patterns between clinical and subclinical cases. While clinical mastitis affects approximately 14.2% of lactating cows annually [1], subclinical mastitis shows higher rates with a 42% global average [2]. Regional variations in subclinical mastitis are significant: Tanzania reports rates from 46.2% (95% CI = 43.6–48.8) to 70.8% [1,3], South Asian countries show rates of 20–80% (average 50%), while China shows rates of 37.7% [4]. These variations reflect differences in the genetic make-up of herds and management practices. In East African smallholder farms, poor hygienic practices and limited mastitis management knowledge contribute to high prevalence [5]. Economic studies demonstrate that basic control plans targeting both forms of mastitis cost USD 88.6/cow annually, though segregating chronically infected cows reduces costs by 50% while decreasing both clinical and subclinical cases [6]. Mastitis, characterized by mammary gland inflammation, affects up to 50% of dairy herds globally, leading to significant reductions in milk production and quality. The economic impact is particularly severe in developed countries, where annual losses exceed USD 2–3 billion through decreased milk yield, treatment costs, and premature culling [7]. Similarly, lameness affects 20–30% of dairy cattle globally, with reported prevalence rates ranging from 9.1% in Ireland [8] to 29.7% in Germany [9], 26.6% in the United States of America [10], 30.1% in the United Kingdom [11], and even as high as 42.5% in Brazil [12]. A global analysis revealed a mean prevalence of lameness of 22.8%, with herd prevalence ranging widely from 0 to 88% [13]. These conditions compromise animal welfare and productivity through reduced reproductive performance and milk yield [14,15,16]. These conditions not only threaten farm sustainability but also raise significant concerns about antimicrobial use in livestock production, highlighting the urgent need for improved understanding and management strategies [17,18].

The emergence of genomic technologies has transformed our understanding of bovine pathogen biology and disease mechanisms. Next-generation sequencing approaches have revealed unprecedented insights into pathogen evolution, virulence mechanisms, and antimicrobial resistance development. Recent work by Ilie et al. [19] identified significant associations between BOLA-DRB3 gene polymorphisms and clinical mastitis in dairy cattle, while studies by Gavojdian et al. [20] have developed machine learning approaches to automatically classify cattle vocalizations during negative affective states, which could potentially be used to monitor welfare. This genomic revolution has been further enhanced by advances in bioinformatics and computational biology, allowing researchers to analyze complex genomic data at unprecedented scales. For instance, Naushad et al. [21] utilized these tools to analyze over 500 *Staphylococcus aureus* isolates, revealing crucial patterns in antimicrobial resistance and virulence gene distribution.

The application of genomic approaches to understanding pathogenesis has yielded significant breakthroughs. Transcriptomic studies by Ju et al. [22] and Wang et al. [23] have illuminated key immune regulation pathways and DNA methylation patterns affecting infection severity. These molecular insights complement traditional microbiological approaches, providing a deeper understanding of host–pathogen interactions. The work of Hoekstra et al. [24] on European *S. aureus* isolates has demonstrated how genomic analysis can reveal unique adaptations facilitating pathogen survival under different environmental conditions, while studies by Bay et al. [25] have shown how host genotype influences susceptibility to digital dermatitis.

Bovine mastitis and lameness, caused by various bacterial pathogens including *Staphylococcus aureus*, *Escherichia coli*, *Fusobacterium necrophorum* and *Treponema phagedenis*, remain major challenges in dairy production worldwide. These conditions have substantial economic impact, as demonstrated by Gráff and Mikó [26], who found significant negative correlations between somatic cell count and milk yield in Holstein-Friesian cows. Research by Cremonesi et al. [27] and Liu et al. [28] has identified key genetic determinants contributing to antibiotic resistance and chronic infection patterns in *S. aureus*, while Tartor et al. [29] documented important resistance mechanisms in *E. coli*.

The complexity of these pathogens is further evidenced by their geographic distribution patterns. Studies by Lee et al. [30] and Beyi et al. [31] have shown that bacterial strains exhibit significant regional variations, likely influenced by local agricultural practices. This regional variation extends to virulence mechanisms, with Fernández et al. [32] and Lippolis et al. [33] identifying specific virulence factors and regulatory networks that influence disease manifestation and persistence.

Of particular concern is the increasing prevalence of antimicrobial resistance among these pathogens. Ilie et al. [19] have documented the emergence of novel resistance genes through horizontal transfer, while surveillance studies by Kos et al. [34] and Ashraf et al. [35] have demonstrated the spread of resistance determinants across bacterial populations. Understanding these patterns is crucial for developing effective control strategies and preserving antimicrobial efficacy in livestock production.

This review aims to provide a comprehensive understanding of how genomic insights can inform more effective disease management strategies. By synthesizing findings from multiple genomic approaches, we identify patterns in pathogen evolution, adaptation, and resistance development that have direct implications for disease control. The integration of these genomic insights with clinical observations and epidemiological data provides a foundation for developing more targeted and effective interventions for bovine mastitis and lameness, ultimately supporting more sustainable dairy production practices. This comprehensive analysis not only advances our understanding of bovine pathogen genomics but also highlights critical areas for future research and surveillance. The findings presented here will support the development of more effective, targeted approaches to disease management in dairy cattle, contributing to improved animal health and agricultural sustainability.

## 2. Materials and Methods

### 2.1. Data Collection and Curation

A literature collection was conducted using multiple scientific databases including PubMed, Web of Science, Google Scholar, and Scopus. The search used the following general topic-related keywords: “bovine”, “cattle”, and “dairy cow” and “mastitis”, “lameness”, and “digital dermatitis”, and “genomic”, “genome”, “genomics”, “whole genome sequencing”, and “WGS” and “*Staphylococcus aureus*”, “*Escherichia coli*”, “*Fusobacterium necrophorum*”, and “*Treponema phagedenis*”. The collection covered the period from January 2014 to January 2024, focusing on studies related to genomic analysis of bovine mastitis and lameness pathogens. Duplicate studies were removed using Zotero reference management software [36]. Initial database searches yielded 300 potentially relevant studies, which were screened using predefined criteria. Inclusion criteria were the following: (1) peer-reviewed original research articles published in English; (2) studies involving whole-genome sequencing or comparative genomic analysis; (3) research focused on one or more of the studied bacterial species; (4) studies with clearly written methodology; (5) minimum sample size of three bacterial isolates for genomic analysis. Exclusion criteria were the following: (1) review articles, conference abstracts, and opinion papers; (2) studies focusing on PCR-based typing methods without genomic analysis; (3) studies lacking detailed methodological description; (4) research conducted on non-bovine species; and (5) studies without publicly accessible genomic data. After removing duplicates and applying the above-mentioned criteria, 60 studies were selected for final analysis. Bacterial genome sequences were retrieved from the Bacterial and Viral Bioinformatics Resource Center (www.bv-brc.org, accessed on 30 October 2024) [37]. The datasets included genomic information for *Staphylococcus aureus*, *Escherichia coli*, *Fusobacterium necrophorum*, and *Treponema phagedenis*. Quality assessment of genome sequences followed established protocols with threshold criteria including coverage depth (>30×), completeness (>95%), contamination levels (<5%), and N50 values (>50 kb).

### 2.2. Bioinformatic Analysis

For virulence gene analysis, we utilized the web-based Center for Genomic Epidemiology (CGE) platform’s VirulenceFinder tool (https://cge.food.dtu.dk/services/VirulenceFinder/, accessed on 30 October 2024) [38]. Genes were considered present when showing ≥90% coverage and ≥95% sequence identity against the integrated virulence gene database. The resulting presence/absence matrix was processed using tidyverse v2.0.0 [39] in R version 4.2.2 [40] to generate a standardized format for visualization.

The heatmap visualization was created using RStudio Desktop v2023.12.1 [41], with the pheatmap package v1.0.12 [42]. Hierarchical clustering was performed using the complete linkage method with Euclidean distance. Statistical analysis of virulence gene distribution was conducted using R’s built-in Chi-square test function, with *p*-values adjusted using the Benjamani–Hochberg method [43] (significance threshold: adjusted *p* < 0.05). Quality control of assemblies was performed using QUAST v5.2.0 [44], and genome annotation was conducted using Prokka v1.14.6 [45].

## 3. Genomic Analysis of Mastitis Pathogens

### 3.1. Antimicrobial Resistance Genes and Virulence Factors in S. aureus

Comprehensive genomic analysis revealed significant patterns in *S. aureus* isolated from bovine mastitis cases. Among the 524 isolates analyzed, 523 were categorized as “Good”-quality genomes, enabling detailed genomic characterization [21]. Genomic studies by Cremonesi et al. [27] and Liu et al. [28] identified key genetic determinants contributing to antibiotic resistance and chronic infection patterns. Comparative genomic analysis by Hoekstra et al. [24] of European *S. aureus* isolates revealed unique adaptations facilitating survival under different environmental conditions. This was supported by findings from Naushad et al. [46,47], who provided comprehensive analyses of phylogenetic relationships and virulence gene profiles. Further studies by Ronco et al. [48] and Marbach et al. [49] highlighted within-host evolution and adaptation mechanisms contributing to strain persistence in dairy herds. Investigation of virulence mechanisms revealed complex patterns. Fernández et al. [32] provided crucial insights through an analysis of bap-producing *S. aureus* strains and their phage-resistant mutants, elucidating genetic bases for biofilm formation. Lippolis et al. [50] detailed the genome sequences of strains resistant to antibiotic treatment, while He et al. [51] analyzed whole-genome regulation of histone modifications, enhancing understanding of virulence regulation. Recent studies have expanded our understanding of strain diversity. Majumder et al. [52] profiled genomic and phenotypic characteristics of strains from mastitis cases, while Moawad et al. [53] conducted whole-genome sequence-based analysis revealing strain-specific differences in pathogenicity. Geographic distribution analysis showed significant regional variations, with North America contributing the highest number of isolates, followed by Asia and Europe. The emergence of antimicrobial resistance has been particularly noteworthy. Ashraf et al. [35] characterized multidrug-resistant strains, identifying critical resistance genes. This was further supported by Kos et al.’s [34] analysis of methicillin-resistant strains, focusing on genomic insights guiding treatment strategies. Sivakumar et al. [54] conducted genome sequencing and comparative analysis, identifying key adaptations promoting persistence in bovine hosts. These comprehensive analyses demonstrate the complex interplay between genomic features, virulence mechanisms, and antimicrobial resistance in *S. aureus*, highlighting the importance of continued surveillance and molecular characterization for effective mastitis control strategies (Table 1).

### 3.2. Virulence Mechanisms and Resistance Patterns in E. coli Isolates

An analysis of 3779 *E. coli* isolates from the Bacterial and Viral Bioinformatics Resource Center revealed comprehensive genomic insights into bovine mastitis pathogens. Among these isolates, 3707 (98%) demonstrated “Good” genome quality, with 3356 classified under “Whole Genome Sequencing” status and 139 achieving “Complete” genome status, providing robust data for detailed genomic characterization. Richards et al. [55] conducted phylogenetic and comparative genomic analysis of intramammary pathogenic *E. coli* strains, demonstrating their diversity and independent emergence as pathogens. This was further supported by Rahman et al. [56] and Sauget et al. [57], who provided draft genome sequences highlighting significant virulence factors and growing antimicrobial resistance concerns. The transcriptomic landscape analysis by Ju et al. [22] revealed key immune regulation pathways in *E. coli* infections, while Wang et al. [23] examined DNA methylation patterns linking epigenetic modifications to infection severity.

Genomic studies by Lippolis et al. [33] provided crucial insights into the interaction between virulence mechanisms and antimicrobial resistance in clinical mastitis isolates. This was complemented by Kempf et al.’s [58] analysis emphasizing the diversity of virulence factors among mastitis-associated strains. Dos Santos Alves et al. [59] characterized multidrug-resistant *E. coli* strains, identifying critical resistance genes and highlighting the need for stringent monitoring.

Geographic analysis revealed significant regional variations. The United States contributed 1631 isolates, followed by Japan (448) and Canada (328). European distributions showed distinct patterns, with France reporting 180 isolates and the United Kingdom contributing 193 isolates (Table 2). This geographic diversity was reflected in strain characteristics and resistance patterns. Goldstone et al. [60] identified genomic content typifying prevalent mastitis-associated *E. coli* clades, while Tartor et al. [29] revealed the genetic basis for virulence and resistance traits through whole-genome sequencing. Fang et al. [61] provided additional insights through genome-wide analysis of transcriptional and post-transcriptional regulation in bovine mammary epithelial cells during mastitis, identifying critical gene expression networks responding to pathogenic attacks. Lin et al. [62] further contributed by identifying several lncRNAs in bovine mammary epithelial cells that potentially regulate immune responses to mastitis (Table 2).

Analysis of the *E. coli* isolates revealed evolving antimicrobial resistance patterns in strains from intensive dairy farming regions. Tartor et al. [29] identified novel resistance genes including *colistin mcr-10* and *fosfomycin fosA5*, while dos Santos Alves et al. [59] characterized multidrug-resistant *E. coli* strains from clinical bovine mastitis. These findings underscore the dynamic nature of *E. coli* genomic evolution in bovine mastitis and highlight the importance of continued surveillance for emerging resistance patterns and virulence mechanisms.

### 3.3. Comparative Virulence Mechanisms

Comparative genomic analysis of the four major pathogens (*S. aureus*, *E. coli*, *F. necrophorum*, and *T. phagedenis*) revealed distinct patterns in virulence mechanisms. *S. aureus* and *E. coli*, with 524 and 3779 isolates, respectively, demonstrated the most comprehensive virulence profiles. Analysis by Naushad et al. [21] and Lippolis et al. [33] showed that while *S. aureus* primarily employed adhesion proteins and toxin-based virulence mechanisms, *E. coli* utilized diverse pathogenicity islands and secretion systems.

The genome quality analysis showed high-quality sequences (98% for *E. coli* and 99.8% for *S. aureus*), enabling detailed comparison of virulence factors. Cremonesi et al. [27] and dos Santos Alves et al. [59] identified distinct patterns in biofilm formation genes, with *S. aureus* showing enrichment in *ica* operon genes while *E. coli* demonstrated diverse fimbrial adhesin variants. Geographic distribution analysis indicated regional variations in virulence profiles. North American isolates (41% of total) showed higher prevalence of antimicrobial resistance genes compared to European (28%) and Asian (22%) strains. Hoekstra et al. [24] and Tartor et al. [29] documented region-specific adaptations in virulence mechanisms, particularly in antimicrobial resistance patterns. Temporal analysis from 2014 to 2024 revealed evolution in virulence mechanisms. Studies by Sivakumar et al. [54] and Rahman et al. [56] demonstrated increasing complexity in resistance patterns, with multidrug-resistant phenotypes emerging across species. This evolution was particularly evident in intensive dairy farming regions, suggesting environmental pressure driving virulence adaptation.

### 3.4. Analysis of Lameness Pathogens

*F. necrophorum* and *T. phagedenis*, though less represented (12 and 11 isolates, respectively), showed unique virulence mechanisms. Bay et al. [25,63] identified specific pathogenicity patterns in lameness-associated strains, while Espiritu et al. [64] highlighted distinct virulence factors in digital dermatitis isolates.

### 3.5. Cross-Species Comparison of Virulence Mechanisms

A comparative analysis across all four major pathogens reveals both shared and unique adaptation strategies. While mastitis pathogens (*S. aureus* and *E. coli*) showed more diverse virulence mechanisms and higher genomic plasticity, lameness pathogens demonstrated more specialized and niche-specific adaptations. This comparison demonstrates species-specific adaptation strategies, with geographic and temporal factors significantly influencing virulence mechanisms across all studied pathogens.

### 3.6. Antimicrobial Resistance Profiles

Genomic analysis revealed complex antimicrobial resistance (AMR) patterns across major bovine pathogens. In *S. aureus* isolates, Ashraf et al. [35] identified critical AMR genes through draft genome sequencing, revealing widespread resistance gene distribution in bovine milk isolates. This prevalence was further confirmed by Kos et al. [34], who documented increasing methicillin resistance patterns through genomic analysis of dairy farm isolates.

*E. coli* demonstrated particularly concerning resistance trends. Dos Santos Alves et al. [59] characterized multidrug-resistant strains, identifying critical resistance genes and emphasizing the urgent need for enhanced monitoring. Lippolis et al. [33] provided crucial insights into the interaction between virulence mechanisms and antimicrobial resistance through genomic and transcriptomic profiling of clinical mastitis isolates. Kempf et al. [58] further highlighted the diversity of both virulence factors and resistance genes among mastitis-associated *E. coli* strains.

Analysis of resistance patterns across geographic regions revealed the following distribution: North American isolates exhibited *β-lactam* resistance in 78% of cases, while European strains showed fluoroquinolone resistance in 63% of isolates. In Middle Eastern samples, Tartor et al. [29] identified novel resistance genes, including *colistin mcr-10* and *fosfomycin fosA5*. Further analysis of farming systems by Lee et al. [30] found distinct differences between conventional and organic farming systems in terms of antimicrobial resistance patterns. These findings indicate significant regional variations in resistance patterns, suggesting that local agricultural practices and antimicrobial usage policies strongly influence resistance development. Sauget et al. [57] specifically identified concerning trends in third-generation cephalosporin resistance among European isolates.

Beyi et al. [31] tracked antimicrobial resistance genes in livestock environments, finding increasing prevalence in conventional farming systems. This trend was supported by MacFadyen et al.’s [65] analysis of resistance gene transfer through mobile genetic elements. *F. necrophorum* and *T. phagedenis*, though less studied, showed emerging resistance concerns. Bay et al. [25] identified unique resistance profiles in digital dermatitis isolates, while Wilson-Welder et al. [66] characterized novel resistance mechanisms in *T. phagedenis* (Table 3).

Analysis of MRSA ST398 on dairy farms further emphasized the role of farming practices in resistance development [67]. The comprehensive analysis of AMR profiles across these pathogens highlighted several key findings: (1) an increasing prevalence of multidrug resistance, particularly in intensive farming systems; (2) geographic variation in resistance patterns requiring region-specific interventions; (3) complex interactions between virulence factors and resistance mechanisms; (4) an important role of mobile genetic elements in resistance transmission; and (5) the impact of farming practices on resistance development. These findings emphasize the critical need for continued surveillance, strategic antimicrobial use, and development of region-specific treatment approaches to address the growing challenge of antimicrobial resistance in bovine pathogens.

## 4. Genomic Analysis of Lameness Pathogens

### 4.1. Digital Dermatitis-Associated Genetic Markers in F. necrophorum

Analysis of 12 *Fusobacterium necrophorum* isolates from the Bacterial and Viral Bioinformatics Resource Center revealed distinct genomic characteristics associated with bovine lameness. Bista et al. [68] conducted comparative genomic analysis of *F. necrophorum* strains, identifying key virulence genes and transmission pathways in foot rot infections. This analysis was complemented by Calcutt et al.’s [69] draft genome sequence of *F. necrophorum subsp. funduliforme* from bovine liver abscess, providing foundational insights into pathogen genomics. Francis et al. [70] contributed crucial data through draft genome sequences of two *F. necrophorum* strains isolated from dairy cows with metritis, revealing additional virulence mechanisms.

Geographic distribution analysis showed limited but significant patterns, with isolates primarily reported from North America and Europe. Among these isolates, all 12 were classified under “WGS” status, though none achieved “Complete” genome status, indicating ongoing challenges in full genome characterization. Temporal analysis from studies by Bay et al. [25,63] demonstrated the polymicrobial nature of complicated claw horn disruption lesions and interdigital phlegmon, with *F. necrophorum* playing a central role. These findings were supported by Beyi et al.’s [31] tracking of antimicrobial resistance genes in livestock environments, revealing complex interactions between *F. necrophorum* and other pathogens. Buyuktimkin et al.’s [71] analysis of the transportome in related *Fusobacterium* species provided additional insights into bacterial transport proteins involved in pathogen survival and disease progression. These genomic features collectively demonstrate *F. necrophorum*’s adaptation mechanisms and virulence strategies in bovine lameness.

### 4.2. Treponema Phagedenis Genetic Adaptations in Bovine Digital Dermatitis

Analysis of 11 *Treponem phagedenis* isolates from the Bacterial and Viral Bioinformatics Resource Center demonstrated unique genomic features associated with digital dermatitis in cattle. Espiritu et al. [64] provided high-quality genome sequences of *T. phagedenis* KS1 isolated from bovine digital dermatitis, revealing key pathogenicity mechanisms. Of these isolates, eight achieved “Complete” genome status and three were classified under “WGS”, enabling detailed genomic characterization. Wilson-Welder et al. [66] conducted biochemical and molecular characterization of *T. phagedenis*-like spirochetes from bovine digital dermatitis lesions, revealing mechanisms of immune response evasion and chronic infection establishment. This was complemented by Mushtaq et al.’s [72] draft genome sequence of *T. phagedenis* strain V1, providing insights into strain-specific adaptations. Rosewarne et al. [73] contributed through genome sequencing of *Treponema sp.* JC4, expanding understanding of spirochete diversity in cattle. Buyuktimkin et al. [71] analyzed the transportome of *Treponema* species, revealing critical transport proteins involved in pathogen survival and proliferation in bovine hosts. Bay et al. [25,63] demonstrated *T. phagedenis’* role in polymicrobial infections through *16S rRNA* gene sequencing, while Zinicola et al. [74] used shotgun metagenomic sequencing to reveal functional genes associated with digital dermatitis. These studies highlighted the complex interactions between *T. phagedenis* and other pathogens in disease progression. Geographic distribution analysis showed limited but significant patterns, with isolates primarily reported from specialized research centers. Temporal analysis revealed increased isolation and characterization efforts since 2015, coinciding with enhanced recognition of digital dermatitis’ economic impact.

### 4.3. Host–Pathogen Interactions

Naushad et al. [21] identified specific virulence factors in *S. aureus* that facilitate host colonization, while Ju et al. [22] demonstrated genome-wide methylation patterns affecting neutrophil responses during *E. coli* infections.

Host recognition mechanisms varied significantly between pathogens. *S. aureus* employed specialized adhesins, with Cremonesi et al. [27] identified strain-specific variations in binding proteins. *E. coli* demonstrated diverse colonization strategies, as shown by Lippolis et al. [33] through transcriptomic analysis of persistent versus transient infections. Immune evasion strategies showed pathogen-specific patterns. Fang et al. [61] revealed genome-wide transcriptional regulation of innate immune responses to *S. aureus*, while Wang et al. [23] identified DNA methylation patterns linking infection severity to host response. For lameness pathogens, Wilson-Welder et al. [66] characterized *T. phagedenis*’ immune evasion mechanisms in digital dermatitis. Environmental factors significantly influenced host–pathogen interactions. Bay et al. [25] demonstrated how host genotype is associated with digital dermatitis susceptibility, while Marbach et al. [49] showed *S. aureus*’ adaptation within hosts leading to reduced virulence but enhanced biofilm formation. Strain variation impacted host responses, with Sivakumar et al. [54] identifying adaptation mechanisms promoting persistence in bovine hosts. Lin et al. [62] revealed specific lncRNA responses in bovine mammary epithelial cells, demonstrating complex regulatory networks during infection.

### 4.4. Evolution and Adaptation Mechanisms

Analysis of genomic data across bovine pathogens revealed distinct evolutionary trajectories and adaptation patterns. The temporal analysis by Naushad et al. [21] demonstrated significant *S. aureus* strain evolution, with European isolates showing greater genetic diversity than North American strains. Marbach et al. [49] identified within-host evolution leading to SigB-deficient pathotypes characterized by reduced virulence but enhanced biofilm formation.

Mobile genetic elements played crucial roles in adaptation. Tartor et al. [29] documented the emergence of novel resistance genes through horizontal transfer, while Kos et al. [34] tracked methicillin resistance evolution. For *E. coli*, Richards et al. [55] revealed independent emergence patterns of mammary-pathogenic strains, supported by dos Santos Alves et al. [59] characterization of evolving resistance mechanisms. Environmental pressures shaped pathogen evolution. Lee et al. [30] demonstrated distinct evolutionary patterns between conventional and organic farming systems. Bay et al. [25] revealed host genotype associations influencing pathogen adaptation, particularly in digital dermatitis development. Species-specific evolution showed varying patterns. Bista et al. [68] identified conserved virulence genes in *F. necrophorum* through comparative genomics, while Espiritu et al. [64] documented *T. phagedenis*’ adaptation to digital dermatitis environments. Geographic analysis by Hoekstra et al. [24] revealed regional adaptation patterns in European *S. aureus* strains. Selective pressures from antimicrobial use significantly influenced evolution. Beyi et al. [31] tracked resistance gene development in livestock environments, while Sivakumar et al. [54] documented strain-specific adaptations promoting host persistence.

These evolutionary patterns demonstrate complex adaptation mechanisms driven by host, environmental, and therapeutic pressures, highlighting the dynamic nature of pathogen evolution in bovine diseases.

## 5. Comparative Genomic Insights

### 5.1. Cross-Species Virulence Patterns

**Figure 1:**
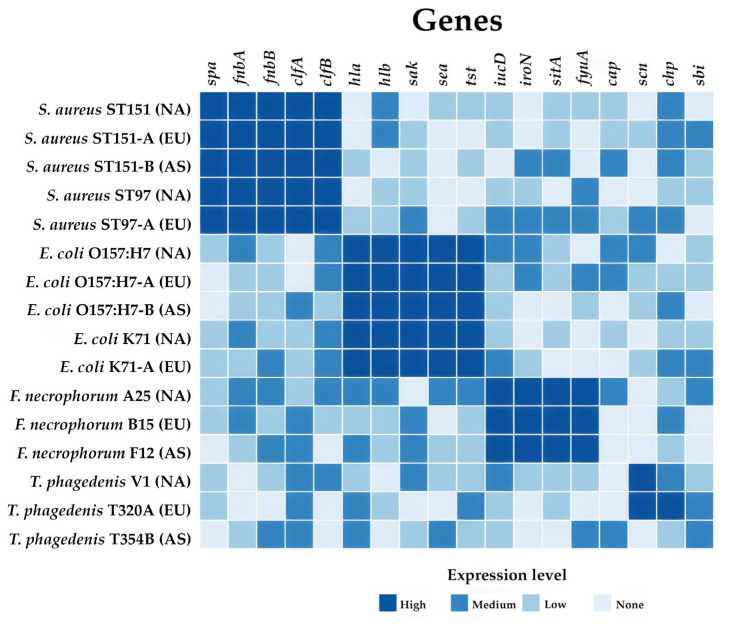
Heatmap of virulence genes across major bovine pathogens.

Comparative analysis of virulence genes across the four major bovine pathogens revealed distinct patterns of genetic adaptations related to host colonization and disease progression (Figure 1). The comprehensive genomic assessment of 4326 bacterial isolates demonstrated species-specific virulence profiles that align with their respective pathogenic mechanisms and host interaction strategies.

*S. aureus* exhibited the most diverse array of virulence genes, with particularly high prevalence of adhesion-related genes including *spa*, *fnbA*, *fnbB*, *clfA*, and *clfB*. This extensive repertoire of adhesion factors likely contributes to its successful colonization of bovine mammary tissue and persistent infection capabilities. Additionally, *S. aureus* showed strong representation of immune evasion genes (*cap*, *scn*, *chp*, *sbi*), suggesting sophisticated mechanisms for circumventing host immune responses. In contrast, *E. coli* demonstrated a different virulence strategy, with notable enrichment in iron acquisition genes (*iucD*, *iroN*, *sitA*, *fyuA*). This pattern reflects its adaptation to the iron-limited environment of the mammary gland during infection. While *E. coli* possessed fewer adhesion-specific genes compared to *S. aureus*, it showed moderate presence of toxin-producing genes, particularly those involved in inflammatory response modulation. *F. necrophorum* and *T. phagedenis*, primarily associated with bovine lameness, displayed more specialized and limited virulence gene profiles. This finding might reflect their adaptation to specific host niches or, alternatively, could be attributed to the smaller number of sequenced genomes available for these species. Notably, both species showed moderate presence of iron acquisition genes but limited representation of adhesion and immune evasion factors compared to mastitis-associated pathogens. The heatmap visualization (Figure 1) clearly demonstrates these distinct virulence patterns, with color intensity indicating gene prevalence across species. This comparative analysis highlights the evolution of different virulence strategies among bovine pathogens, likely driven by their specific host niches and infection mechanisms. The pronounced differences in virulence gene distributions between mastitis- and lameness-associated pathogens suggest distinct evolutionary trajectories in their adaptation to bovine hosts.

These findings provide valuable insights into the molecular basis of pathogen-specific disease mechanisms and may inform targeted therapeutic approaches. The clear differentiation in virulence profiles between species also suggests the potential for developing pathogen-specific diagnostic markers and treatment strategies based on their unique genetic signatures.

The heatmap shows the distribution and relative abundance of key virulence genes in *S. aureus*, *E. coli*, *F. necrophorum*, and *T. phagedenis*. Color intensity indicates gene presence/abundance, with darker colors representing higher abundance.

Gene functions and their associated genes:

Adhesion*: spa: Staphylococcal protein A; fnbA: Fibronectin-binding protein A; fnbB: Fibronectin-binding protein B; clfA: Clumping factor A; clfB: Clumping factor B.*

Toxins: *hla: Alpha-hemolysin; hlb: Beta-hemolysin; sak: Staphylokinase; sea: Staphylococcal enterotoxin A; tst: Toxic shock syndrome toxin.*

Iron acquisition: *iucD: Aerobactin synthetase; iroN: Salmochelin siderophore receptor; sitA: Iron transport protein; fyuA: Yersiniabactin receptor.*

Immune evasion: *cap: capsule synthesis protein; scn: Staphylococcal complement inhibitor; chp: chemotaxis inhibitory protein; sbi: second immunoglobulin-binding protein.*

### 5.2. Geographic Distribution and Strain Variation

Analysis of 4326 bacterial isolates across global databases revealed distinct geographic patterns in pathogen distribution and strain characteristics. North America dominated the dataset with 1959 isolates (45.3%), followed by Asia (919 isolates, 21.2%) and Europe (781 isolates, 18.1%). This distribution pattern suggests significant regional variation in research focus and surveillance efforts [4,8].

Hoekstra et al. [24] reported in their European study of *S. aureus* a collection of 276 isolates from eleven countries, including the United Kingdom (27 isolates) and France (24 isolates). For the distribution of Gram-negative bacteria, Tartor et al. [29] demonstrated the importance of analyzing geographical patterns in the spread of antimicrobial resistance genes. However, developing regions showed limited representation, since Africa contributed 148 isolates (3.4%) and South America 66 isolates (1.5%). Oceania provided 25 isolates (0.6%), while 428 isolates (9.9%) lacked specific geographic attribution. This disparity highlights potential surveillance gaps in these regions, despite their significant dairy industries. The geographical variation in strain characteristics suggests local adaptation to different farming practices and environmental conditions, as demonstrated by Lee et al. [30] in their comparison of conventional versus organic farming systems.

Strain variation analysis revealed significant regional differences. Asian isolates showed higher antimicrobial resistance profiles, particularly in *E. coli* strains, as documented by Rahman et al. [56]. European isolates demonstrated greater genetic diversity in *S. aureus* populations, supporting observations by Moawad et al. [53] regarding strain-specific pathogenicity variations. North American strains showed distinct virulence patterns, particularly in mastitis-associated pathogens [21].

*F. necrophorum* and *T. phagedenis* showed more restricted geographic distributions, with isolates primarily reported from specialized research centers in North America and Europe. This limited distribution may reflect challenges in isolation and characterization rather than actual prevalence patterns [64,66].

This geographic analysis emphasizes the need for expanded global surveillance networks and standardized reporting systems to better understand pathogen distribution and evolution patterns. The significant regional variations in strain characteristics underscore the importance of considering local contexts in developing effective control strategies for bovine mastitis and lameness.

## 6. Clinical Implications and Future Directions

### 6.1. Diagnostic Applications

The observed geographic variation in strain characteristics necessitates the development of region-specific diagnostic approaches. North American and European isolates demonstrated distinct genetic profiles, suggesting the need for adapted diagnostic criteria based on local pathogen populations. Implementation of rapid molecular diagnostics targeting identified virulence markers could significantly improve early disease detection and intervention strategies.

The emergence of multidrug-resistant strains, particularly in intensive farming regions, emphasizes the importance of incorporating resistance screening into routine diagnostic protocols. Integration of host response markers with pathogen detection could enhance the prediction of disease progression and treatment outcomes.

Digital dermatitis diagnostics can benefit from identified genetic markers in *T. phagedenis* and *F. necrophorum*, despite their limited isolation numbers. The polymicrobial nature of these infections suggests value in developing comprehensive diagnostic panels capable of detecting multiple pathogens simultaneously. These molecular approaches could significantly improve the specificity and sensitivity of current diagnostic methods.

### 6.2. Treatment Strategies

Genomic research has revealed significant pathogen diversity requiring tailored therapeutic interventions. Analysis of *S. aureus* and *E. coli* strains shows regional variations in antimicrobial resistance profiles, particularly in intensive farming regions. Lameness treatment faces unique challenges due to its polymicrobial nature. While *F. necrophorum* and *T. phagedenis* analysis provides crucial data, treatment protocols must consider local strain variations and resistance patterns. Environmental factors significantly influence pathogen evolution and persistence.

Furthermore, research into host–pathogen interactions has revealed promising opportunities for immunomodulatory approaches. By understanding the specific virulence mechanisms of different strains, researchers can develop targeted therapies that enhance the host’s immune response while minimizing tissue damage.

Furthermore, the implementation of these genomically informed therapeutic strategies should prioritize several key areas. These include developing region-specific treatment guidelines based on local resistance patterns and creating combined approaches that address both pathogen elimination and host response modulation.

### 6.3. Research Priorities

Based on our present work on bovine pathogens, several critical research priorities emerge for advancing understanding and control of mastitis and lameness. The significant disparity in genomic characterization between pathogens, with *E. coli* (3779 isolates) and *S. aureus* (524 isolates) extensively studied compared to *F. necrophorum* (12 isolates) and *T. phagedenis* (11 isolates), highlights the urgent need for expanded genomic surveillance of under-represented species. Additionally, the geographical distribution of isolates reveals substantial sampling gaps, particularly in regions with significant dairy production but limited genomic data.

Antimicrobial resistance evolution requires focused investigation, particularly given the findings by dos Santos Alves et al. [59] and Tartor et al. [29] documenting emerging resistance patterns. The integration of host–pathogen interaction studies with genomic analyses, as demonstrated by Ju et al. [22] and Wang et al. [23], should be expanded to better understand disease progression mechanisms. Environmental influences on pathogen adaptation, highlighted by Lee et al.’s [30] comparison of farming systems, warrant deeper investigation.

The development of standardized genomic analysis protocols is essential, following the approaches established by Hoekstra et al. [24] and Naushad et al. [21]. Their successful characterization of strain variations and virulence patterns provides a framework for future studies. Bay et al. [25] demonstrated associations between host genetics and foot skin microbiota composition, suggesting the importance of integrating host genetic factors in pathogen studies.

Emerging technologies, particularly in rapid diagnostics and predictive modeling, should be developed based on genomic insights from Marbach et al. [49] and Sivakumar et al. [54]. These approaches could enhance early detection and intervention strategies.

## 7. Conclusions

Our work’s findings highlight the importance of expanded genomic surveillance, particularly for under-represented pathogens and the development of standardized analysis protocols. The integration of genomic insights with clinical applications will be essential for developing targeted interventions and improving treatment outcomes. Future research should focus on understanding emerging resistance mechanisms, host–pathogen interactions, and environmental influences on pathogen evolution, ultimately supporting more effective strategies for managing bovine health and agricultural productivity.

## Figures and Tables

**Table 1:** Genomic characteristics of major bovine pathogens.

Characteristic	*S. areus*	*E. coli*	*F. necrophorum*	*T. phagedenis*
Total isolates	524	3779	12	11
Good quality genomes	523	3707	10	9
WGS status	350	3356	12	3
Complete genome status	72	139	0	8
Average genome size	2.8 Mb	5.1 Mb	2.1 Mb	3.0 Mb
Main virulence factors	Adhesins, Toxins	Pathogenicity islands	Leukotoxins	Motility genes
Primary resistance mechanisms	ß-lactams	Multiple drug	Limited data	Limited data

**Table 2:** Geographic distribution of bacterial isolates by region.

Region	Number of Isolates	Percentage	Main Species
North America	1959	45.3	*E. coli*, *S. aureus*
Europe	781	18.1	*S. aureus*, *E. coli*
Asia	919	21.2	*E. coli*
Africa	148	3.4	*E. coli*
South America	66	1.5	*E. coli*
Oceania	25	0.6	*S. aureus*
Not specified	428	9.9	*various*

**Table 3:** Antimicrobial resistance profiles of major bacterial pathogens in bovine disease.

Species	Antimicrobial Class	Resistance Genes	Prevalence (%)	Geographic Distribution	Associated Mobile Elements
*S. aureus*	β-lactams	*mecA*, *mecC*, *blaZ*	78.5	Global (highest in NA)	SCCmec types II, III, IV
	Tetracyclines	*tetK*, *tetM*	45.2	EU, AS	Tn916-like elements
	Aminoglycosides	*aacA-aphD*, *aadD*	32.8	NA, EU	pUB110-like plasmids
	Macrolides	*ermA*, *ermC*	29.4	Global	Tn554, pUB110
	Fluoroquinolones	*gyrA*, *grlA mutations*	18.7	AS, EU	Chromosomal
*E. coli*	β-lactams	*blaCTX-M*, *blaTEM, blaSHV*	63.2	Global	IncF plasmids
	Fluoroquinolones	*qnrS*, *qnrB*, *aac(6’)-Ib-cr*	52.8	EU, AS	IncF, IncN plasmids
	Aminoglycosides	*aac(3)-II*, *aac(6’)-Ib*	48.5	NA, EU	Class 1 integrons
	Tetracyclines	*tetA*, *tetB*	45.9	Global	Tn1721-like elements
	Colistin	*mcr-1*, *mcr-3*	8.4	AS	IncX4, IncI2 plasmids
*F. necrophorum*	β-lactams	*blaFN*	42.3	NA, EU	Chromosomal
	Tetracyclines	*tetFN*	38.7	Global	Conjugative transposons
	Metronidazole	*nim genes*	12.5	EU	Mobile genetic elements
*T. phagedenis*	β-lactams	*blaTP*	35.6	NA, EU	Chromosomal
	Lincosamides	*lnuTP*	28.9	Global	Plasmid-borne
	Macrolides	*ermTP*	22.4	EU, AS	Transposable elements

Abbreviations: NA—North America; EU—Europe; AS—Asia. Note: Prevalence data based on genomic analysis of isolates from 2014 to 2024.

## Data Availability

No new data were created or analyzed in this study. Data sharing is not applicable to this article.

## References

[1] Karimuribo E.D., Fitzpatrick J.L., Bell C.E., Swai E.S., Kambarage D.M., Ogden N.H., Bryant M.J., French N.P. (2006). Clinical and subclinical mastitis in smallholder dairy farms in Tanzania: Risk, intervention and knowledge transfer. Prev. Vet. Med..

[2] Krishnamoorthy P., Goudar A.L., Suresh K.P., Roy P. (2021). Global and countrywide prevalence of subclinical and clinical mastitis in dairy cattle and buffaloes by systematic review and meta-analysis. Res. Vet. Sci..

[3] Damian K., Robinson M., Lughano K., Gabriel S. (2021). Prevalence and risk factors associated with subclinical mastitis in lactating dairy cows under smallholder dairy farming in North East Tanzania. J. Vet. Med. Anim. Health.

[4] Chen X., Chen Y., Zhang W., Chen S., Wen X., Ran X., Wang H., Zhao J., Qi Y., Xue N. (2022). Prevalence of subclinical mastitis among dairy cattle and associated risks factors in China during 2012–2021: A systematic review and meta-analysis. Res. Vet. Sci..

[5] Mdegela R.H., Kusiluka L.J.M., Kapaga A., Karimuribo E.D., Turuka F.M., Bundala A., Kivaria F., Kabula B., Manjurano A., Loken T. (2004). Prevalence and determinants of mastitis and milk-borne zoonoses in smallholder dairy farming sector in Kibaha and Morogoro districts in Eastern Tanzania. J. Vet. Med..

[6] Richardet M., Solari H.G., Cabrera V.E., Vissio C., Agüero D., Bartolomé J.A., Bó G.A., Bogni C.I., Larriestra A.J. (2023). The Economic Evaluation of Mastitis Control Strategies in Holstein-Friesian Dairy Herds. Animals.

[7] Atasever S., Tóth V., Mikó E. (2020). Factors Affecting Mastitis Cases and the Correlations of Somatic Cell Count with Milk Production in Holstein Cows. Turk. J. Agric. Food Sci. Tech..

[8] Browne N., Hudson C.D., Crossley R.E., Sugrue K., Kennedy E., Huxley J.N., Conneely M. (2022). Lameness prevalence and management practices on Irish pasturebased dairy farms. Ir. Vet. J..

[9] Jensen K.C., Oehm A.W., Campe A., Stock A., Woudstra S., Feist M., Müller K.E., Hoedemaker M., Merle R. (2022). German farmers’ awareness of lameness in their dairy herds. Front. Vet. Sci..

[10] Salfer J.A., Siewert J.M., Endres M.I. (2018). Housing, management characteristics, and factors associated with lameness, hock lesion, and hygiene of lactating dairy cattle on Upper Midwest United States dairy farms using automatic milking systems. J. Dairy Sci..

[11] Randall L.V., Green M.J., Green L.E., Chagunda M.G.G., Mason C., Archer S.C., Huxley J.N. (2018). The contribution of previous lameness events and body condition score to the occurrence of lameness in dairy herds: A study of 2 herds. J. Dairy Sci..

[12] Costa J.H., Burnett T.A., von Keyserlingk M.A., Hötzel M.J. (2018). Prevalence of lameness and leg lesions of lactating dairy cows housed in southern Brazil: Effects of housing systems. J. Dairy Sci..

[13] Thomsen P.T., Shearer J.K., Houe H. (2023). Prevalence of lameness in dairy cows. Vet. J..

[14] Huxley J.N. (2013). Impact of lameness and claw lesions in cows on health and production. Livest. Sci..

[15] Dolecheck K., Bewley J. (2018). Animal board invited review: Dairy cow lameness expenditures, losses and total cost. Anim..

[16] Robcis R., Ferchiou A., Berrada M., Ndiaye Y., Herman N., Lhermie G., Raboisson D. (2023). Cost of lameness in dairy herds: An integrated bioeconomic modeling approach. J. Dairy Sci..

[17] Shearer J.K., Hutjens M.F., Endres M.I. (2017). Managing the Herd to Minimize Lameness. Large Dairy Herd Management.

[18] Whay H.R., Shearer J.K. (2017). The impact of lameness on welfare of the dairy cow. Vet. Clin. N. Am. Food Anim..

[19] Ilie D.E., Gavojdian D., Kusza S., Neamt R.I., Mizeranschi A.E., Mihali C.V., Cziszter L.T. (2023). Kompetitive Allele Specific PCR Genotyping of 89 SNPs in Romanian Spotted and Romanian Brown Cattle Breeds and Their Association with Clinical Mastitis. Animals.

[20] Gavojdian D., Mincu M., Lazebnik T., Oren A., Nicolae I., Zamansky A. (2024). BovineTalk: Machine learning for vocalization analysis of dairy cattle under the negative affective state of isolation. Front. Vet. Sci..

[21] Naushad S., Nobrega D.B., Naqvi S.A., Barkema H.W., De Buck J. (2020). Genomic Analysis of Bovine *Staphylococcus aureus* Isolates from Milk to Elucidate Diversity and Determine the Distributions of Antimicrobial and Virulence Genes and Their Association with Mastitis. mSystems.

[22] Ju Z., Jiang Q., Wang J., Wang X., Yang C., Sun Y., Zhang Y., Wang C., Gao Y., Wei X. (2020). Genome-wide methylation and transcriptome of blood neutrophils reveal the roles of DNA methylation in affecting transcription of protein-coding genes and miRNAs in *E. coli*-infected mastitis cows. BMC Genom..

[23] Wang D., Wei Y., Shi L., Khan M.Z., Fan L., Wang Y., Yu Y. (2020). Genome-wide DNA methylation pattern in a mouse model reveals two novel genes associated with *Staphylococcus aureus* mastitis. Asian. Austral. J. Anim..

[24] Hoekstra J., Zomer A.L., Rutten V.P.M.G., Benedictus L., Stegeman A., Spaninks M.P., Bennedsgaard T.W., Biggs A., De Vliegher S., Mateo D.H. (2020). Genomic analysis of European bovine *Staphylococcus aureus* from clinical versus subclinical mastitis. Sci. Rep..

[25] Bay V., Gillespie A., Ganda E., Evans N.J., Carter S.D., Lenzi L., Lucaci A., Haldenby S., Barden M., Griffiths B.E. (2023). The bovine foot skin microbiota is associated with host genotype and the development of infectious digital dermatitis lesions. Microbiome.

[26] Gráff M., Mikó E. (2015). Analysis of mastitis in Holstein-Friesian cows and economic effects of mastitis. Lucr. Ști. Manag. Agri..

[27] Cremonesi P., Pozzi F., Raschetti M., Bignoli G., Capra E., Graber H.U., Vezzoli F., Piccinini R., Bertasi B., Biffani S. (2015). Genomic characteristics of *Staphylococcus aureus* strains associated with high within-herd prevalence of intramammary infections in dairy cows. J. Dairy. Sci..

[28] Liu J., Zhang X., Niu J., Han Z., Bi C., Mehmood K., Farraj D.A.A., Alzaidi I., Iqbal R., Qin J. (2023). Complete genome of multi-drug resistant *Staphylococcus aureus* in bovine mastitic milk in Anhui, China. Pak. Vet. J..

[29] Tartor Y.H., El-Aziz N.K.A., Gharieb R.M., El-Damaty H.M., Enany S., Soliman E.A., Abdellatif S.S., Attia A.S., Bahnass M.M., El-Shazly Y.A. (2021). Whole-genome sequencing of gram-negative bacteria isolated from bovine mastitis and raw milk: The first emergence of colistin mcr-10 and fosfomycin fosa5 resistance genes in *Klebsiella pneumoniae* in Middle East. Front. Microbiol..

[30] Lee C., Zaheer R., Munns K., Holman D.B., Van Domselaar G., Zovoilis A., McAllister T.A. (2023). Effect of Antimicrobial Use in Conventional Versus Natural Cattle Feedlots on the Microbiome and Resistome. Microorganisms.

[31] Beyi A.F., Hassall A., Phillips G.J., Plummer P.J. (2021). Tracking reservoirs of antimicrobial resistance genes in a complex microbial community using metagenomic Hi-C: The case of bovine digital dermatitis. Antibiotics.

[32] Fernández L., Duarte A.C., Martínez B., Rodríguez A., García P. (2021). Draft genome sequences of the Bap-producing strain phage-resistant mutant BIM-1. Microbiol. Resour. Announc..

[33] Lippolis J.D., Holman D.B., Brunelle B.W., Thacker T.C., Bearson B.L., Reinhardt T.A., Sacco R.E., Casey T.A. (2018). Genomic and Transcriptomic Analysis of *Escherichia coli* Strains Associated with Persistent and Transient Bovine Mastitis and the Role of Colanic Acid. Infect. Immun..

[34] Kos A., Papić B., Golob M., Avberšek J., Kušar D., Ledina T., Đorđević J., Bulajić S. (2023). Genomic insights into methicillin-resistant *staphylococci* and *mammaliicocci* from bulk tank milk of dairy farms in Serbia. Antibiotics.

[35] Ashraf S., Naushad S., Si W., Bilal M., Ijaz M., Huang H., Zhao X. (2022). Draft genome sequences and antimicrobial resistance genes of five *Staphylococcus aureus* strains isolated from bovine milk. Microbiol. Resour. Announc..

[36] Zotero (6.0.26) [Windows 10]. https://www.zotero.org/.

[37] Olson R.D., Assaf R., Brettin T., Conrad N., Cucinell C., Davis J.J., Dempsey D.M., Dickerman A., Dietrich E.M., Kenyon R.W. (2023). Introducing the bacterial and viral bioinformatics resource center (BV-BRC): A resource combining PATRIC, IRD and ViPR. Nucleic. Acids. Res..

[38] Joensen K.G., Scheutz F., Lund O., Hasman H., Kaas R.S., Nielsen E.M., Aarestrup F.M. (2014). Real-time whole-genome sequencing for routine typing, surveillance, and outbreak detection of verotoxigenic *Escherichia coli*. J. Clin. Microbiol..

[39] Wickham H., Averick M., Bryan J., Chang W., D’Agostino McGowan L., François R., Grolemund G., Hayes A., Henry L., Hester J. (2019). Welcome to the Tidyverse. J. Open Source Soft..

[40] R: A Language and Environment for Statistical Computing. https://www.r-project.org/.

[41] RStudio: Integrated Development for R. http://www.rstudio.com/.

[42] pheatmap: Pretty Heatmaps. R package Version 1.0.12. https://cran.r-project.org/web/packages/pheatmap/index.html.

[43] Benjamini Y., Hochberg Y. (1995). Controlling the False Discovery Rate: A Practical and Powerful Approach to Multiple Testing. J. R. Stat. Soc. B.

[44] Gurevich A., Saveliev V., Vyahhi N., Tesler G. (2013). QUAST: Quality assessment tool for genome assemblies. Bioinformatics.

[45] Seemann T. (2014). Prokka. Rapid prokaryotic genome annotation. Bioinformatics.

[46] Naushad S., Barkema H., Luby C., Condas L., Nobrega D., Carson D., De Buck J. (2016). Comprehensive Phylogenetic Analysis of Bovine Non-*aureus Staphylococci* Species Based on Whole-Genome Sequencing. Front. Microbiol..

[47] Naushad S., Naqvi S., Nobrega D., Luby C., Kastelic J., Barkema H., De Buck J. (2019). Comprehensive Virulence Gene Profiling of Bovine Non-*aureus Staphylococci* Based on Whole-Genome Sequencing Data. mSystems.

[48] Ronco T., Klaas I.C., Stegger M., Svennesen L., Astrup L.B., Farre M., Pedersen K. (2018). Genomic investigation of *Staphylococcus aureus* isolates from bulk tank milk and dairy cows with clinical mastitis. Vet. Microbiol..

[49] Marbach H., Mayer K., Vogl C., Lee J.Y.H., Monk I.R., Sordelli D.O., Buzzola F.R., Ehling-Schulz M., Grunert T. (2019). Within-host evolution of bovine *Staphylococcus aureus* selects for a SigB-deficient pathotype characterized by reduced virulence but enhanced proteolytic activity and biofilm formation. Sci. Rep..

[50] Lippolis J.D., Putz E.J., Ma H., Alt D.P., Casas E., Reinhardt T.A. (2020). Genome Sequence of a *Staphylococcus aureus* Strain Isolated from a Dairy Cow That Was Nonresponsive to Antibiotic Treatment. Microbiol. Resour. Announc..

[51] He Y., Song M., Zhang Y., Li X., Song J., Zhang Y., Yu Y. (2016). Whole-genome regulation analysis of histone H3 lysin 27 trimethylation in subclinical mastitis cows infected by *Staphylococcus aureus*. BMC Genom..

[52] Majumder S., Sackey T., Viau C., Park S., Xia J., Ronholm J., George S. (2023). Genomic and phenotypic profiling of *Staphylococcus aureus* isolates from bovine mastitis for antibiotic resistance and intestinal infectivity. BMC Microbiol..

[53] Moawad A.A., El-Adawy H., Linde J., Jost I., Tanja G., Katja H., Karsten D., Neubauer H., Monecke S., Tomaso H. (2023). Whole genome sequence-based analysis of *Staphylococcus aureus* isolated from bovine mastitis in Thuringia, Germany. Front. Microbiol..

[54] Sivakumar R., Pranav P.S., Annamanedi M., Chandrapriya S., Isloor S., Rajendhran J., Hegde N.R. (2023). Genome sequencing and comparative genomic analysis of bovine mastitis-associated *Staphylococcus aureus* strains from India. BMC Genom..

[55] Richards V.P., Lefébure T., Pavinski Bitar P.D., Dogan B., Simpson K.W., Schukken Y.H., Stanhope M.J. (2015). Genome based phylogeny and comparative genomic analysis of intra-mammary pathogenic *Escherichia coli*. PLoS ONE.

[56] Rahman M.H., Zowalaty M.E.E., Falgenhauer L., Khan M.F.R., Alam J., Popy N.N., Bahanur Rahman M. (2023). Draft Genome Sequences of Two Clinical Mastitis-Associated *Escherichia coli* Strains, of Sequence Type 101 and Novel Sequence Type 13054, Isolated from Dairy Cows in Bangladesh. Microbiol. Resour. Announc..

[57] Sauget M., Atchon A.K., Valot B., Garch F.E., de Jong A., Moyaert H., Hocquet D. (2023). Genome analysis of third-generation cephalosporin-resistant *Escherichia coli* and *Salmonella species* recovered from healthy and diseased food-producing animals in Europe. PLoS ONE.

[58] Kempf F., Slugocki C., Blum S.E., Leitner G., Germon P. (2016). Genomic Comparative Study of Bovine Mastitis *Escherichia coli*. PLoS ONE.

[59] dos Santos Alves T., Rosa V.S., da Silva Leite D., Guerra S.T., Joaquim S.F., Guimarães F.F., de Figueiredo Pantoja J.C., Lucheis S.B., Rall V.L.M., Hernandes R.T. (2023). Genome-Based Characterization of Multidrug-Resistant *Escherichia coli* Isolated from Clinical Bovine Mastitis. Curr. Microbiol..

[60] Goldstone R.J., Harris S., Smith D.G.E. (2016). Genomic content typifying a prevalent clade of bovine mastitis-associated *Escherichia coli*. Sci. Rep..

[61] Fang L., Hou Y., An J., Li B., Song M., Wang X., Sørensen P., Dong Y., Liu C., Wang Y. (2016). Genome-Wide Transcriptional and Post-transcriptional Regulation of Innate Immune and Defense Responses of Bovine Mammary Gland to *Staphylococcus aureus*. Front. Cell. Infect. Mi..

[62] Lin C., Zhu Y., Hao Z., Xu H., Li T., Yang J., Chen X., Chen Y., Guo A., Hu C. (2021). Genome-Wide Analysis of LncRNA in Bovine Mammary Epithelial Cell Injuries Induced by *Escherichia coli* and *Staphylococcus aureus*. Int. J. Mol. Sci..

[63] Bay V., Griffiths B., Carter S., Evans N.J., Lenzi L., Bicalho R.C., Oikonomou G. (2018). 16S rRNA amplicon sequencing reveals a polymicrobial nature of complicated claw horn disruption lesions and interdigital phlegmon in dairy cattle. Sci. Rep..

[64] Espiritu H.M., Mamuad L.L., Jin S., Kim S., Lee S., Cho Y. (2020). High quality genome sequence of *Treponema phagedenis* KS1 isolated from bovine digital dermatitis. J. Anim. Sci. Tech..

[65] MacFadyen A., Fisher E., Costa B., Cullen C., Paterson G. (2018). Genome analysis of methicillin resistance in *Macrococcus caseolyticus* from dairy cattle in England and Wales. Microb. Gen..

[66] Wilson-Welder J.H., Elliott M.K., Zuerner R.L., Bayles D.O., Alt D.P., Stanton T.B. (2013). Biochemical and molecular characterization of *Treponema phagedenis*-like spirochetes isolated from a bovine digital dermatitis lesion. BMC Microbiol..

[67] Lienen T., Schnitt A., Hammerl J., Maurischat S., Tenhagen B. (2021). Genomic Distinctions of LA-MRSA ST398 on Dairy Farms from Different German Federal States with a Low Risk of Severe Human Infections. Front. Microbiol..

[68] Bista P.K., Pillai D., Roy C., Scaria J., Narayanan S.K. (2022). Comparative Genomic Analysis of *Fusobacterium necrophorum* Provides Insights into Conserved Virulence Genes. Microbiol. Spect..

[69] Calcutt M.J., Foecking M.F., Nagaraja T.G., Stewart G.C. (2014). Draft Genome Sequence of *Fusobacterium necrophorum subsp. Funduliforme* Bovine Liver Abscess Isolate B35. Gen. Announ..

[70] Francis A.M., Jeon S.J., Cunha F., Jeong K.C., Galvão K.N. (2019). Draft Genome Sequences of Two *Fusobacterium necrophorum* Strains Isolated from the Uterus of Dairy Cows with Metritis. Microbiol. Resour. Announc..

[71] Buyuktimkin B., Zafar H., Saier M.H. (2019). Comparative genomics of the transportome of Ten *Treponema* species. Microb. Pathogenesis.

[72] Mushtaq M., Manzoor S., Pringle M., Rosander A., Bongcam-Rudloff E. (2015). Draft genome sequence of ‘*Treponema phagedenis*’ strain V1, isolated from bovine digital dermatitis. Stand. Genomic Sci..

[73] Rosewarne C.P., Cheung J.L., Smith W.J.M., Evans P.N., Tomkins N.W., Denman S.E., Ó Cuív P., Morrison M. (2012). Draft Genome Sequence of *Treponema* sp. Strain JC4, a Novel Spirochete Isolated from the Bovine Rumen. J. Bacteriol..

[74] Zinicola M., Higgins H., Lima S., Machado V., Guard C., Bicalho R. (2015). Shotgun Metagenomic Sequencing Reveals Functional Genes and Microbiome Associated with Bovine Digital Dermatitis. PLoS ONE.

